# Assessment of Basal Cell Carcinoma Using Dermoscopy and High Frequency Ultrasound Examination

**DOI:** 10.3390/diagnostics12030735

**Published:** 2022-03-18

**Authors:** Ioana-Alina Halip, Dan Vâţă, Laura Statescu, Paul Salahoru, Adriana Ionela Patraşcu, Doinita Temelie Olinici, Bogdan Tarcau, Ioana-Adriana Popescu, Madalina Mocanu, Anne-Marie Constantin, Maria Crisan, Ilarie Brihan, Alin Codrut Nicolescu, Laura Gheuca-Solovastru

**Affiliations:** 1 Department of Dermatology, “Grigore T. Popa” University of Medicine and Pharmacy, 700115 Iasi, Romania; danvata@yahoo.com (D.V.); laura.statescu@yahoo.com (L.S.); oana.manolache@yahoo.com (I.-A.P.); lsolovastru13@yahoo.com (L.G.-S.); 2 Dermatology Clinic, “St. Spiridon” County Emergency Clinical Hospital, 700115 Iasi, Romania; patrascuai@yahoo.com (A.I.P.); doinitzaganceanu@yahoo.com (D.T.O.); bogdan.tarcau@yahoo.com (B.T.); 3 Thoracic Surgery Department, “Grigore T. Popa” University of Medicine and Pharmacy, 700115 Iasi, Romania; paul_salahoru@yahoo.com; 4 Department of Oral Dermatology, “Grigore T. Popa” University of Medicine and Pharmacy, 700115 Iasi, Romania; drmadalinamocanu@yahoo.com; 5 Histology Department, Faculty of Medicine, “Iuliu Hațieganu” University of Medicine and Pharmacy, 400000 Cluj-Napoca, Romania; annemarie_chindris@yahoo.com (A.-M.C.); mcrisan7@yahoo.com (M.C.); 6 Department of Dermatology, Faculty of Medicine and Pharmacy, University of Oradea, 410073 Oradea, Romania; 7 Roma Medical Center for Diagnosis and Treatment, 011773 Bucharest, Romania

**Keywords:** basal cell carcinoma, dermoscopy, high frequency ultrasound examination

## Abstract

Basal cell carcinoma (BCC) is the most common form of cutaneous neoplasia in humans, and dermoscopy may provide valuable information for histopathological classification of BCC, which allows for the choice of non-invasive topical or surgical therapy. Similarly, dermoscopy may allow for the identification of incipient forms of BCC that cannot be detected in clinical examination. The importance of early diagnosis using the dermoscopy of superficial BCC forms is proven by the fact that despite their indolent clinical appearance, they can be included in high-risk BCC forms due to the rate of postoperative recurrence. Nodular pigmentary forms of BCCs present ovoid gray-blue nests or multiple gray-blue dots/globules associated with arborized vessels, sometimes undetectable on clinical examination. The management of BCC depends on this, as pigmentary forms have been shown to have a poor response to photodynamic therapy. High frequency ultrasound examination (HFUS) aids in the diagnosis of BCC with hypoechoic tumour masses, as well as in estimating tumour size (thickness and diameter), presurgical margin delineation, and surgical planning. The examination is also useful for determining the invasion of adjacent structures and for studying local recurrences. The use of dermoscopy in combination with HFUS allows for optimisation of the management of the oncological patient.

## 1. Introduction

Basal cell carcinoma (BCC) is a malignant nonmelanocytic tumour that forms by the proliferation of slowly growing epidermal basal layer cells on the tumour-free integument or on other pre-existing lesions, showing in situ malignancy manifested by invasiveness and recurrence. It is the most common form of cutaneous neoplasia in humans and in terms of incidence globally, there is an upward trend in the number of new cases, with the American Dermatology Association (ADA) estimating an annual number of 2 million cases, thus exceeding the incidence of other types of neoplasia [1,2,3].

Skin neoplasia is the most common human neoplasia and often presents ethical issues [4].

BCC is seen in people of all races and skin types, with dark-skinned individuals rarely affected, and is most often seen in fair-skinned individuals (phototype 1 or phototype 2) [5]. In terms of gender distribution, the reported incidence is higher in men, with a male to female ratio of approximately 1.5–2:1, and is probably due to increased recreational and occupational exposure to the sun, although these differences become less significant with changes in lifestyle [6]. Anatomically, for periocular BCC tumours, the incidence is equal in men and women. Age-adjusted incidence rates for palpebral BCC in men and women are 16.9 and 12.4 cases per 100,000 persons per year, respectively [7].

## 2. Dermoscopy of Basal Cell Carcinoma

The well-documented value of dermoscopy for the diagnosis of BCC is gaining a pivotal role in tumour management. Clinicians’ therapeutic algorithm for BCC includes several surgical methods, as well as non-surgical modalities [8]. The choice of an appropriate treatment depends on several factors, including histopathological subtype, presence of pigmentation or ulceration, tumour thickness, anatomical location, and presence of residual lesions or recurrence. It has been shown that dermoscopy provides valuable information for several of the above parameters [8].

The dermoscopic appearance of the tumour is influenced by factors such as gender, age, and phototype. Studies report a higher frequency of superficial BCC on the trunk and lower limbs of females, while most nodular BCC occur in the cephalic and cervical region of males [9,10]. Pigmentation is present in more than 50% of dark phototype tumours, while less than 10% of BCCs in light phototype individuals are pigmented [11]. The pattern of multiple tumours in the same patient usually shows a repetitive dermoscopic pattern [12].

Dermoscopic findings in basal cell carcinoma tumour lesions indicate the following:

1. Large-diameter, bright-red, prominent-looking arborizing vessels (Figure 1) and fine superficial telangiectasias corresponding to dilated dermal neovascularization vessels. Arborizing telangiectasias represent the most characteristic vascular pattern in nodular BCC. On the one hand, these vessels are large-diameter vessels (more than 0.2 mm) [13] and they branch at irregular intervals into increasingly thin capillaries. Their colour is bright red, have abrupt border cut-offs and are in sharp focus. Arborizing telangiectasias seen using dermoscopy are located immediately below the epidermis, on the surface of the tumour and throughout the tumour. On the other hand, nontumoral vessels located in the dermis are generally pink and appear out of focus due to the effect of the dispersion of light through the dermal connective tissue [14].
  In approximately 90% of superficial BCCs in sharp focus, short fine telangiectasias are found. They consist of small tortuous vessels, measuring less than 1 mm in length and displaying little or no branching. In sclerodermiform BCCs, the vessels are generally thinner, more diffuse, and have less branching [14].

**Figure 1:**
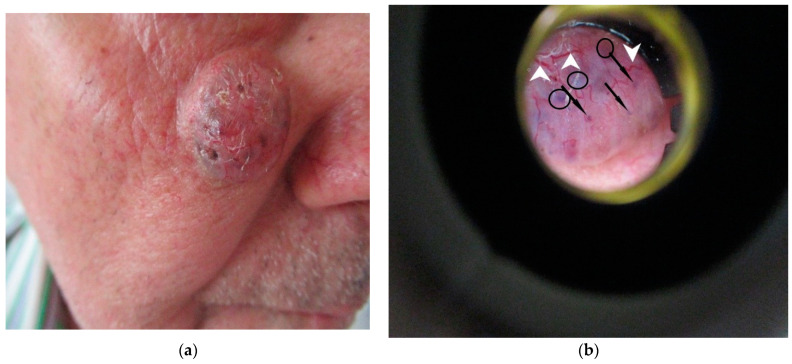
Nodular pigmented BCC: clinical aspect (**a**) and dermoscopic (Dermlite^®^) appearance showing small bluish-gray dot-like structures (indicated my black arrows) and bluish-brown globules (indicated by black circles) accompanied by arborized vessels (indicated by white arrows) (**b**) (own collection).
2. Brown or blue pigmented structures of multiple shapes:
  - small focused gray, blue, or brown spots (with diameter smaller than 0.1 mm) representing free pigment deposition along the dermo−epidermal junction, and/or melanophages and/or small aggregates of pigmented neoplastic cells in the papillary and reticular dermis [13]. Similarly, dermoscopy can identify large blue-brown-gray globules (with diameter equal with 0.1 mm or bigger than 0.1 mm) (Figure 1). Histologically, they represent small, roundish tumour nests with central pigmentation, localized to the papillary dermis and/or reticular dermis;
  - ovoid blue-gray nests corresponding to large nests of tumour cells with pigment aggregates invading the dermis;
  - foliaceous elements with a “maple leaf-like” appearance are gray, brown, or bluish homogeneous structures that are not associated with the pigment network, and their morphology is reminiscent of leaves, with brown or gray/blue extensions in the periphery, always without connection to the pigmentary network or adjacent pigmentary areas (Figure 2). “Maple leaf-like” structures represent multifocal tumour nests with pigment aggregates connected to each other by lobular extensions, located mainly in the epidermis and less frequently located in the dermis;

**Figure 2:**
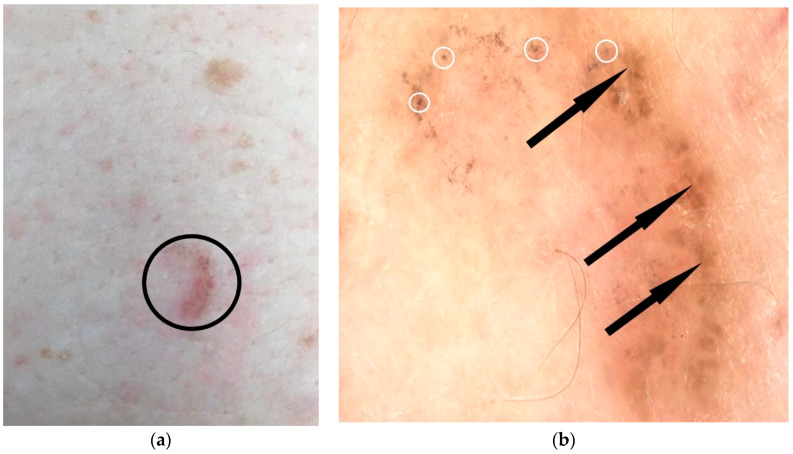
Pigmentary BCC: clinical aspect (indicated by black circle) (**a**) and videodermatoscopic appearance using Visiomed microDERM AG^®^ (×30) targeting “maple leaf-like” elements (indicated by black arrows) which are brown homogeneous structures that are not associated with the pigment network, and their morphology is reminiscent of leaves, with brown extensions in the periphery, always without connection to the pigmentary network or adjacent pigmentary areas; similarly, “spoke−wheel-like” pigmentary structures (indicated by white circles) as radially distributed brown structures, with a central axis and with brown radial projections, are identified (**b**) (own collection).
  - “spoke−wheel-like structures” are radially distributed blue-gray structures, with a central axis and with brown, gray, or black radial projections (Figure 2). Histologically, they correspond to pigmented clusters of cells originating from the basal layers or from the epidermis, with multiple extensions into dermis;
  - irregular concentric structures of variable colour (blue, gray, brown, or black) and with a darker centre, constituting the precursors of the “spoke−wheel-like” structures;
3. Small multiple erosions or ulcerations commonly covered by haematic crusts (Figure 3a,b);

**Figure 3:**
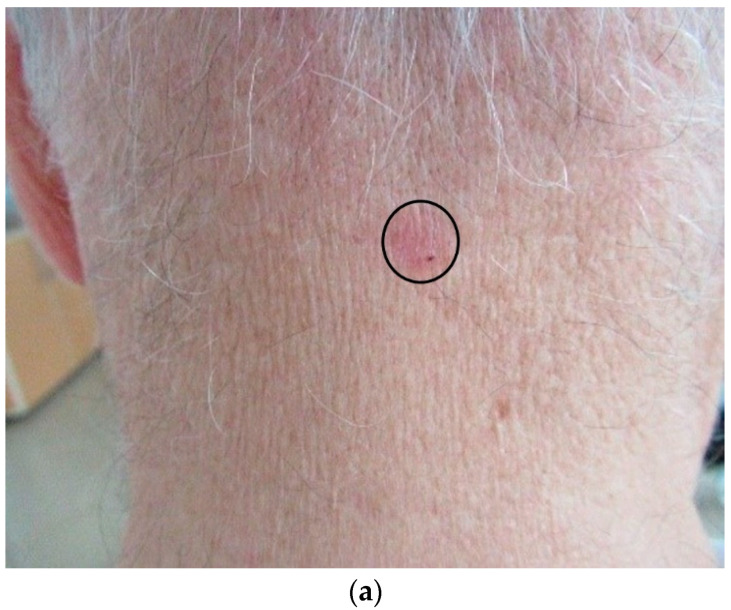

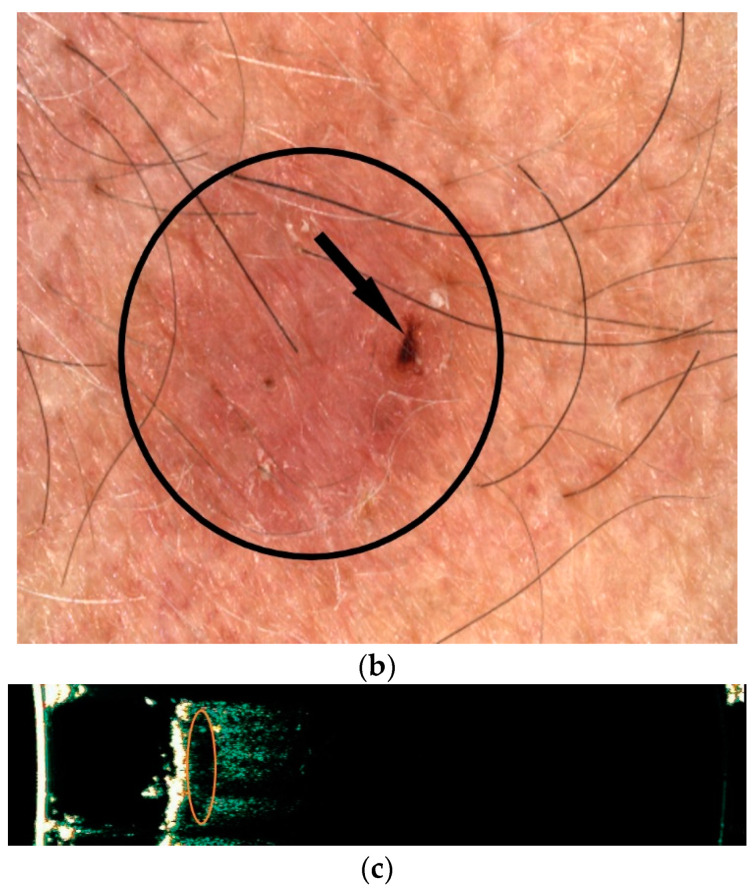
Example of superficial BCC examination: the clinical appearance of an oval, pink-translucent, ulcerated tumour located on the photoexposed cervical area (indicated by black circle) (**a**), videodermoscopic aspect (using Visiomed microDERM AG^®^—×15) showing a small ulceration (indicated by a black arrow) (**b**), HFUS examination (using Dermascan C^®^ 20 MHz) with evidence of a subepidermal, well-demarcated, linear hypoechoic mass (indicated by orange circle) representing the superficial basal cell carcinoma (**c**) (own collection).
4. shiny white-red structureless areas appear as translucent to opaque white to red areas, and their histologic correlation is diffuse dermal fibrosis or fibrotic tumoral stroma;
5. white crystalline streaks (chrysalis) visible only in polarized light dermoscopy signify the presence of collagenous stroma or fibrosis in the dermis [15,16].

Dermoscopy allows for the identification of incipient forms of BCC that cannot be detected upon clinical examination [14]. Dermoscopy, in addition to diagnosing BCC tumours, offers the possibility of histopathological classification of the BCC examined, which allows for the choice of non-invasive topical or surgical therapy. Thus, superficial forms do not show thick arboreal vessels, as shown in Figure 1, but show linear vessels or fine telangiectasias with few branches, accompanied by multiple small erosions or white-pink areas in non-pigmented forms. Superficial pigmented BCC forms show concentric gray or light brown “spoke−wheel-like” structures or leafy elements with a “maple leaf” appearance. The identification of bluish-gray nests excludes the diagnosis of superficial BCC and confirms BCC forms with a dermal component [15].

In the differential diagnosis, actinic keratoses located on the rest of the body usually show a non-specific pattern, including an irregular shape, white or yellowish scales, concentric yellow structures, and erythema, sometimes with red dots corresponding to vessels, and white rosette-like structures or white lines under polarized light [17]. Superficial BCCs show bright pinkish-white structures and fine telangiectasias, unlike Bowen’s disease, which shows cluster and glomerular pattern vessels accompanied by scales. For psoriasis, the significant features consist of a vascular pattern with red dots dotted on a light erythematous background [18].

The importance of early diagnosis using dermoscopy of superficial BCC forms has been proven by the fact that despite their indolent clinical appearance, they can be included in high-risk BCC forms due to the rate of postoperative recurrence [19,20]. This is due to the tendency to extend further into the periphery than the tumour margins allow upon clinical examination, leading to incomplete excisions and an increased recurrence rate. Therefore, the first line of therapy consists of non-ablative immunomodulatory therapies (imiquimod) and photodynamic therapy [21]. In contrast, nodular forms have a good response rate to surgical treatment and topical therapies are less effective [8].

Nodular nonpigmented BCCs, which are also the most common forms, typically present arborized vessels and ulcerations, whereas the pigmentary forms of BCC present ovoid gray-blue nests or multiple gray-blue dots/globules associated with arborized vessels, sometimes undetectable upon clinical examination. “Spoke−wheel-like” are well-circumscribed radial projections, blue or gray showing a dark brown, black, or blue central axis (Figure 2b). Histopathologically, they represent pigmented clusters of cells originating from the basal layers or from the epidermis, with multiple extensions into the dermis. The rate of their postoperative appearance is significant, and radially distributed blue-gray structures are typically specific for BCC diagnosis. “Spoke−wheel” areas represent a highly specific BCC feature and can be seen in all subtypes, but more frequently in superficial BCC. In contrast, “maple-leaf” structures present as gray, brown, or bluish homogeneous structures that are not associated with the pigment network and their morphology is similar to leaves (“maple leaf areas”). In nodular nonpigmented BCC, “spoke−wheel-like “ or “maple-leaf” structures at the periphery of lesions are uncommon. Moreover, dermoscopic features in nonpigmented forms allow for differentiation from squamous cell carcinomas, amelanotic melanomas, and other nonpigmented tumours.

**Figure 4:**
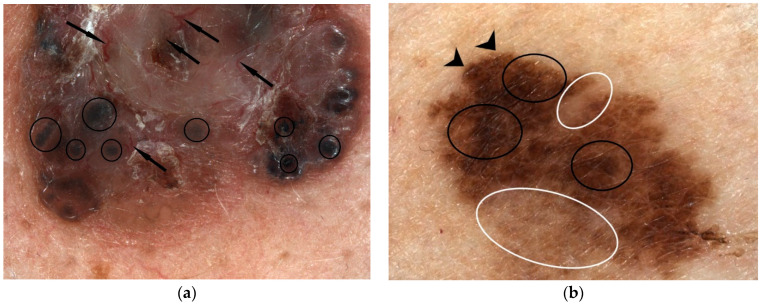
Pigmentary nodular BCC at videodermoscopic examination (using Visiomed microDERM AG^®^ × 15 magnification) showing bright red, sharp in focus and large arborizing vessels (indicated by black arrows), and blue gray globules (indicated by black circles), with no presence of the pigmentary network (**a**). Extensive superficial malignant melanoma (Clark III Breslow 1.1 mm, pT1b Nx, Pn0) with videodermoscopic appearance (using Visiomed microDERM AG^®^ ×30 magnification), which indicates an asymmetric tumour lesion, with a longitudinal diameter greater than 6 mm (D1/D2 = 13.4/6.7 mm), with the presence of an atypical pigmented network with irregular blotches (indicated by black circles), with radial streaming (parallel linear extension at the periphery indicated by black arrows) and regression, with the white area lighter than the surrounding skin (indicated by white circles) (**b**) (own collection).

Nodular pigmentary BCC forms in the differential diagnosis with nevi-like melanocytic tumours have absent pigmentary network, with the presence of pigmentary structures such as dots and/or blue-gray globules (Figure 4a). The differentiation of pigmented or superficial forms with a discrete milky veil, pigmented globules, or uncharacteristic vessels from malignant melanoma is aimed at excluding signs of melanocytic lesions with the pigmentary network [22,23].

The main dermoscopic features of melanoma include the following:

1. Asymmetry in colour and structure and variegated colours, showing an atypical pigmented black, brown, or gray network defined by lines that are thicker than holes, with irregular meshes situated on the periphery (Figure 4b) and the lines stop abruptly. Dots represent the accumulation of melanin in the epidermis and a small nest of melanocytes in the lower part of the dermis, basal membrane, or papillary dermis. Globules represent nests of melanocytes in the papillary dermis, on the tips of rete ridges, and in the lower epidermis. In nevi the dots and globules have uniform size, colour, and shape and are distributed symmetrically, whereas in melanoma, atypical dots and globules have different sizes, shapes, and colours (Figure 4b), and are usually located at the periphery of the lesion. The morphological definition of blotches indicates black, brown, and/or gray pigmented areas with an irregular shape or distribution. Associated histopathological changes consist of hyperpigmentation throughout the epidermis and/or upper dermis. Regression structures are either white areas lighter than the surrounding skin or small, gray/blue dots with a “peppering” appearance. Regression is determined by thickened papillary dermis with fibrosis areas, or by combinations of both and/or variable amounts of melanophages [24].
2. Melanoma specific structures:
  - streaks are horizontal spreading of melanoma and histopathology shows nests of atypical melanocytes at the dermo−epidermal junction. Streaks include radial streaming (parallel linear extension at the periphery of the lesion with focally and asymmetrically pattern in spreading melanoma) and pseudopodes (finger-like projections or dark pigment at the periphery with small knobs at their tips, and are connected or with blotches or with pigment network).
  - A blue-white veil is represented by irregular blue pigmentation with an overlying white “ground glass” haze, and histological examination indicates clusters of pigmented melanocytes or melanin in the dermis with ortokeratosis of the epidermis.
  - crystalline structures—short white shiny lines that might be parallel or vertical to one to the other. This criterion may be seen only with polarized dermoscopy [24].
3. Vascular structures may show polymorphic vessels like pinpoint or dotted vessels (appear in dermoscopy as very small red dots with diameters of 0.01–0.02 mm and they correspond to the tips of short, vertically arranged capillaries in lesions with smaller diameters), linear irregular, corkscrew (appearing as balls of wool that resemble renal glomeruli), or milky red areas [25].

In contrast, in BCCs, the main vessels are large-diameter (0.2 mm or larger) branching into smaller, thinner branches in non-homogenous terminal capillaries with a diameter of 10 µm. Vascular structures in nodular melanoma diameters of over 3 mm mimic BCC at first glance, but the vessels in these tumours display a more homogenous arborization [25].

The diagnostic accuracy of Menzies dermoscopy (absence of pigmentary network and presence of six positive criteria: arborised vessels, presence of ulceration, large blue-gray ovoid nests, blue-gray dots and globules, and “maple leaf” and “spoke−wheel-like“ structures) has achieved a sensitivity of 97% and a specificity of 92% and 93% for differentiating pigmented BCC forms from melanoma and nevi, respectively [16].

BCCs may exhibit equivocal dermoscopic patterns, without typical dermoscopic criteria, simulating malignant melanoma. Non-facial BCCs simulating malignant melanomas have been described in the literature presenting an atypical network, blue-white veil, irregular dots/globules, irregular streaks, and an atypical vascular pattern. Moreover, the presence of atypical vessels and irregular streaks, specifically for lower limb BCCs, has been reported [26]. Mateo et al., in a retrospective, multicentric comparative study of atypical, non-facial BCC and melanoma, observed 12 “trump” dermoscopic features they used to develop a proposed score for differential diagnosis among BCCs mimicking malignant melanoma (≥1 seven-point checklist criteria) and malignant melanoma with BCC features (with at least one BCC criteria at dermoscopy). The proposed score (sensitivity 94.1% and specificity 79.5%) included regression structures (+3), irregular dots/globules (+3), irregular blotches (+2), irregular streaks (+2), white-red structureless areas (+1), white streaks (+1), “spoke−wheel” areas (−1), in-focus dots (−1), multiple blue-gray globules (−1), arborizing vessels (−2), concentric structures (−3), and “maple leaf-like” areas (−3). The score had a total cut-off for melanoma larger than 2, and for atypical BCC equal or smaller than 2. A total of 146 basal cell carcinomas and 76 melanomas were included and the results showed that an atypical vascular pattern was common to most lesions (74.5%). The lack of pigmented features and the observation of atypical vessels was the most confounding patterns. Their results support that an atypical vascular pattern should be considered a shared feature of both malignant melanoma and atypical BCC, and may improve diagnostic accuracy and confidence in daily clinical practice [26]. A previous retrospective study of facial and non-facial excised BCCs with histopathological diagnosis investigated the variability and significance of dermoscopic features of atypical BCCs. The findings showed that dermoscopic features suggestive of melanocytic lesions were observed in 40.6% of BCCs and were significantly increased in heavily pigmented BCCs [22].

Infiltrative and sclerodermiform BCC forms also show dermoscopically branched vessels, usually finer and with fewer branches compared to the classical nodular BCC vessels. In contrast to the overall pink, translucent colour of nodular BCC, infiltrative BCC often shows white/red areas structurally, whereas the underlying fibrosis of sclerodermiform BCC results in a whitish background. Pinkus fibroepithelioma is an unusual variant of BCC, characterized dermoscopically by a white-pink background colour with fine arborized vessels in the centre and punctate vessels in the periphery [15,23].

Metatypical or baso-squamous cell carcinomas have the simultaneous presence of at least one feature of both BCC and invasive squamous cell carcinoma (SCC). Thus, they may have unfocused (peripheral) arborizing vessels, blue-gray blotches such as in BCC and white astructural areas, scales, ulcerations, hematic crusts, and red spots in keratin masses such as in SCC [27]. Out of focus vessels are seen secondary to acanthosis from hyperkeratotic epidermis [28]. The most common findings of baso-squamous cell carcinomas are peripheral arborizing vessels of those similar to BCC, while keratinization findings such as keratin masses, ulceration, and white structureless areas are the most common non-vascular features similar to SCC [28,29]. White structures represented by white circles and central masses of keratin are useful clues for SCC. The central mass of keratin appears as amorphous, yellow-white to light-brown areas without any recognizable structure. Similarly, in SCC, targetoid hair follicles present keratotic plugs within the follicular openings of the skin, mostly over a white structureless area. BCC nevoid form is an unusual variant of the tumour, usually associated with patients with Gorlin−Goltz syndrome. Dermoscopy in these cases may indicate brownish pigmentation similar to that seen in nevi, and also blue-gray dots, globules, or nests, often combined with arborizing vessels [15].

Dermoscopy is a useful method for the detection of pigmented BCC, even in infraclinical forms. This is explained by the fact that small and few pigmentary foci may be insufficient to determine obvious clinical pigmentation. Thus, it has been shown that 30% of non-pigmentary BCC forms upon clinical examination actually represented histopathological forms of pigmented BCC [30]. The management of BCC depends on this, as pigmentary forms have been shown to have a poor response to photodynamic therapy, regulations that have been introduced in photodynamic therapy guidelines [31]. The low efficacy is attributed to melanin acting competitively in light absorption, decreasing the response rate to treatment [31,32]. Cases in which pigmented forms of BCC cannot be clinically recognized can lead to errors in the choice of appropriate treatment and decreased therapeutic efficacy [30]. Radially distributed blue-gray structures are typically specific for BCC diagnosis. Spoke−wheel areas represent a highly specific BCC feature and can be seen in all subtypes, but more frequently in superficial BCC [32]. “Spoke−wheel-like” areas, “maple leafe-like areas”, and concentric structures are identified in superficial tumours and, when present in non-superficial BCC, they are usually seen at the periphery of the lesion, whereas the nodular part often presents blue-gray nests or globules [32].

Traditional surgical excision is the treatment of choice in most BCC tumours. For the prevention of incomplete excision, the most reliable method is Mohs surgery, which is considered the optimal treatment for aggressive tumour subtypes (e.g., morpheaform BCC) and for BCC localized on the face [33]. However, using the recommended 3 mm lateral excision margin, conventional surgery has been associated with recurrence rates of up to 17% [33,34]. Dermoscopy provides a better assessment of the true extent of the tumour than clinical examination, and allows for a more accurate estimate of the surgical margins required, helping to minimise the recurrence rate. Carducci et al. suggested that the margins of healthy perilesional integument can be defined by the absence of well-known dermatoscopic criteria of BCC, such as ovoid nests, blue-gray globules, or maple leaf-like structures [35]. Discrimination of tumour vessels from those of the healthy integument can be based on the blurred appearance and dark reddish-purple colour of vessels in the photo-aged integument, as opposed to the focused, bright red vessels of the tumour [32].

Dermoscopy plays an essential role in monitoring photodynamic therapy, as the clinical appearance often does not allow for estimation of the residual lesions. In such situations, clinicians may resort to several possibilities: the conservative (“wait and see”) one whereby they wait for the post-therapy response, either perform a biopsy or opt for a new therapeutic course, or other more aggressive therapeutic methods. These situations can lead to a risk of suboptimal or, on the contrary, overly aggressive treatment, resulting in prolonged comorbidities and increased economic costs. The absence of dermoscopic criteria of superficial BCC after non-ablative procedures safely predicts complete histopathological clearance, while the presence of arborizing vessels, ulceration, and/or pigmented structures represents tumour persistence or recurrence [36]. The detection of structureless white/red areas and/or superficial fine telangiectasias justifies close monitoring to recognize early recurrence [37].

## 3. High-Frequency Ultrasonography of Basal Cell Carcinoma

Ultrasonography aids in the diagnosis of BCC, with lower density (hypoechoic) tumour masses replacing (more hyperechoic) collagen, as well as in estimating the tumour size (thickness and diameter), pre-surgical margin delineation, and surgical planning [38]. The examination is also useful for determining the invasion of adjacent structures (cartilage and/or bone), as well as for assessing response to non-surgical treatments and studying local recurrences. Basal cell carcinoma can be distinguished from other tumour types by the appearance of a hypoechoic mass (Figure 3c), with hyperechogenic areas often in the interior due to corneous cysts, microcalcifications, or nests of apoptotic cells called “flower cotton”, an aspect that distinguishes it from squamous cell carcinoma and melanoma, being well demarcated, irregular in outline, and usually located in the dermis but with possible extension into the underlying tissues [39].

The presence of seven or more hyperechogenic spots within the lesion has been associated with histological subtypes of BCC having a high risk of recurrence, such as micronodular, infiltrative, or morpheaform and metatypical forms [40,41].

Morphology obtained sonographically can reduce the number of incomplete or conversely too wide excisions, which could lead to aesthetic or functional problems, and in the case of non-invasive therapy, is useful for monitoring therapeutic efficacy [42,43]. Examination of post-surgical lesions in follow-up has an important role in detecting recurrences.

One publication [44] correlated ultrasonographic assessment of superficial and nodular BCC with histopathological measurements of post excision tumours (with fixed 4 mm margins), determining that HFUS was able to accurately indicate which tumours had subclinical extension beyond the 4 mm margins in 48 out of 50 cases. Another study, which examined the role of HFUS in improving the surgical accuracy of Mohs micrographic surgery for nonmelanocytic carcinomas (regardless of histologic subtype), found that HFUS was more sensitive when determining subclinical extension in tumours with an area greater than 1.74 cm^2^ [43].

Many studies have confirmed the high correlation between tumour thickness obtained by means of the histopathological examination and ultrasonography of up to 88% and 96% (correlation coefficient) [45]. This is particularly important in the planning of surgery. Good knowledge of the limits of tumour infiltration can contribute to an increased percentage of radical treatments. It is known, however, that the ultrasonographically determined tumour thickness is slightly greater than the histologically determined thickness, and this difference is due to tissue shrinkage during histological preparation and the presence of inflammatory infiltrate around the tumour [46].

Ultrasound allows for a multimodal approach of BCC, which completes clinical and histological examinations, improves the therapeutic management, and may assess the therapeutic efficacy and tumoral prognosis [47].

Thus, a limitation of ultrasonographic examination may occur in inflammatory infiltrates associated with basal cell carcinomas or melanomas, which may create hypoechoic extension leading to an overestimation of the tumour size. The reticular dermal extension of BCC cannot be visualized with 20 MHz HFUS, requiring frequencies in the 13-15 MHz range. If a tumour extends into the subcutaneous connective tissue, delineation can be difficult, as its appearance is also hypoechoic and therefore HFUS may not be suitable for the assessment of deeper tumours [48].

Two sonographic artifacts have been reported in BCC lesions: the first is termed “angles at the bottom” of the lesion, which is produced by perilesional inflammatory components, and the second is termed “blurry tumour”, which is generated by a large presence of sebaceous glands that are isoechogenic with the primary lesion and may decrease the definition of the tumour margins [41,49].

Tumour thickness determined by means of HFUS may also be useful for selecting patients for non-surgical treatment. In one study [50], they identified in 65 BCC tumours smaller than 2 mm in size (for which photodynamic therapy was performed), and for tumours larger than 3 mm, ultrasonographically guided laser-ablative diode ablation therapy in combination with photodynamic therapy was performed (with a response rate of 100% tumour clearance at the 6-month follow-up).

## 4. Conclusions

Using high-frequency ultrasonography in the examination of basal cell carcinomas, the dermatologist can complement the clinical examination and obtain additional clues for histopathological subtype differentiation, margin delineation, and tumour size assessment.

The use of dermoscopy in combination with high frequency ultrasonography allows for optimisation of the management of the oncological patient and the establishment of prognostic factors, with the classification of basal cell carcinomas into either a low or high risk of recurrence, by quantifying the ultrasonographic and dermatoscopic examinations.
